# The Puzzling Problem of Cardiolipin Membrane-Cytochrome c Interactions: A Combined Infrared and Fluorescence Study

**DOI:** 10.3390/ijms22031334

**Published:** 2021-01-29

**Authors:** Francesca Ripanti, Almerinda Di Venere, Mariangela Cestelli Guidi, Martina Romani, Alessandra Filabozzi, Marina Carbonaro, Maria Cristina Piro, Federica Sinibaldi, Alessandro Nucara, Giampiero Mei

**Affiliations:** 1 Department of Physics, Sapienza University of Rome, P.le A. Moro 5, 00185 Rome, Italy; francesca.ripanti@uniroma1.it; 2 Department of Experimental Medicine, Tor Vergata University of Rome, Via Montpellier 1, 00133 Rome, Italy; divenere@med.uniroma2.it (A.D.V.); piro@med.uniroma2.it (M.C.P.); sinibaldi@med.uniroma2.it (F.S.); mei@med.uniroma2.it (G.M.); 3 INFN-Laboratori Nazionali di Frascati, Via Enrico Fermi 40, 00044 Frascati, Italy; mariangela.cestelliguidi@lnf.infn.it (M.C.G.); martina.romani@lnf.infn.it (M.R.); 4 Department of Physics, Tor Vergata University of Rome, Via della Ricerca Scientifica 1, 00133 Rome, Italy; alessandra.filabozzi@roma2.infn.it; 5 Council for Agricultural Research and Economics (CREA), Research Centre for Food and Nutrition, Via Ardeatina 546, 00178 Rome, Italy; marina.carbonaro@crea.gov.it

**Keywords:** protein-membrane binding, membrane disorder, cytochrome c, cardiolipin

## Abstract

The interaction of cytochrome c (cyt c) with natural and synthetic membranes is known to be a complex phenomenon, involving both protein and lipid conformational changes. In this paper, we combined infrared and fluorescence spectroscopy to study the structural transformation occurring to the lipid network of cardiolipin-containing large unilamellar vesicles (LUVs). The data, collected at increasing protein/lipid ratio, demonstrate the existence of a multi-phase process, which is characterized by: (i) the interaction of cyt c with the lipid polar heads; (ii) the lipid anchorage of the protein on the membrane surface; and (iii) a long-distance order/disorder transition of the cardiolipin acyl chains. Such effects have been quantitatively interpreted introducing specific order parameters and discussed in the frame of the models on cyt c activity reported in literature.

## 1. Introduction

The interactions of cell membranes with soluble peripheral proteins give rise to interesting phenomena that induce relevant changes in both molecular structures. Typically, such heterogeneous systems are characterized by a high degree of complexity, due to the conflicting properties featuring the two counterparts: The polar, hydrophilic peripheral residues lying on the outer shell of such proteins, and the high hydrophobic environment existing inside the membrane layers [1]. Necessarily, the onset of the interaction involves the membrane surface, i.e., a boundary region in which even more difficulties arise, due to the heterogeneous composition of the phospholipid polar heads and to the presence of hydration shells surrounding cells and organelles. Generally, two classes of binding of peripheral proteins to biological membranes can be highlighted, namely specific associations (such as those involving lipid anchors) or non-specific contacts, which include hydrogen bond, hydrophobic, and electrostatic interactions. The latter ones, particularly sensitive to pH, are rather weak in physiological conditions, allowing the reversible attachment/detachment of small positively charged proteins to the negative surface of double layers, such as the inner membranes of mitochondria. Cytochrome c (cyt c) represents one of the most studied peripheral proteins in the last four decades; its peculiar properties and behavior are still challenging both for experimentalists and theoreticians. At first considered as simple electron-carrier between complexes III and IV of the mitochondria respiratory chain, this small protein (12 kDa molecular weight) has been recognized to play a key role in activating apoptotic cell death signals [2,3], and also to act as a scavenger of radical oxygen species in healthy cells [4]. Cyt c functions are also related to the association with cardiolipin (CL)-containing membranes, a process that fosters additional protein capabilities such as the strong peroxidase activity [5], required to permeabilize the mitochondria membrane and to initialize the apoptotic cascade [6]. Therefore, the mechanism of interaction between this protein, in native and nano-aggregate form, and the cell membrane is still one of the most debated issues. In spite of a large number of experiments conceived to estimate the equilibrium binding and the kinetics of this association, details of the molecular mechanisms remain uncertain, as too many effects simultaneously occur, not trivially governed by a number of variables (membrane composition, ionic strength, and presence of nucleotides, to mention a few). Previous experiments stated that both electrostatic and non-electrostatic contacts stabilize the protein-membrane complex [7,8,9,10], probably in synergy with a strong anchorage of one CL acyl chain into a hydrophobic cyt c pocket [11,12,13]. The balance among these forces seems to be strictly dependent on the number of protein molecules lying on the vesicle surface [14]. Indeed, the protein binding (and the protein crowding) is responsible for both tertiary structural changes of the protein [9,10,15,16] and its penetration into the lipid bilayer [17]. The membrane, in turn, is influenced by the binding process as well, with different effects ranging from lipid lateral domain assembly [18], to non-bilayer structures [19,20], and pore generation [20].

The present work was aimed at highlighting structural and conformational changes of the lipid network upon cyt c binding, at different values of the protein/lipid concentration (hereafter indicated as *ρ* = [cyt c]/[CL]). These changes, interpreted in the framework of ordered-to-disordered transitions, as those occurring in molecular systems, were mainly investigated by Attenuated Total Reflection InfraRed spectroscopy (ATR-IR), which provides the absorption features from the molecular vibrational modes [21]. Such approach allowed to monitor three kinds of different processes occurring when cyt c interacts with CL-containing large unilamellar vesicles (LUVs): (i) the formation of disordered segments in the phospholipids acyl-chains, as revealed by changes in the stretching vibrational bands of the CH_2_ and CH_3_ groups; (ii) the tight binding of cyt c to CL, due to the insertion of an acyl chain into the protein hydrophobic core [11]; and (iii) the effects of protein-membrane association on the lipid polar heads, diagnostic of O-P-O vibrational modes.

A set of parallel experiments was also carried out using an independent technique, namely Fluorescence Resonance Energy Transfer (FRET), at low *ρ* values (<0.015). The overall data analysis provided evidence of a heterogeneous set of binding curves, suggesting the existence of multiple phenomena, taking place at different *ρ* values. This finding was discussed in the frame of a recently proposed multiple step binding model [9,10], taking also into account the differences observed by Oellerich and co-workers [14] between the peripheral and partial penetration mode of binding, at increasing cyt c concentrations.

## 2. Results

### 2.1. ATR-IR Spectroscopy

Attenuated Total Reflectance measurements were performed on cyt c-LUV drops at different *ρ* ratios. Two spectral regions have been considered in the data analysis, namely that of the CH_2_, CH_3_, and CH=CH modes, at 2830–3030 cm^−1^, and that of the phosphate bands at 900–1300 cm^−1^.

#### 2.1.1. Spectra of the Acyl Chains

**Figure 1:**
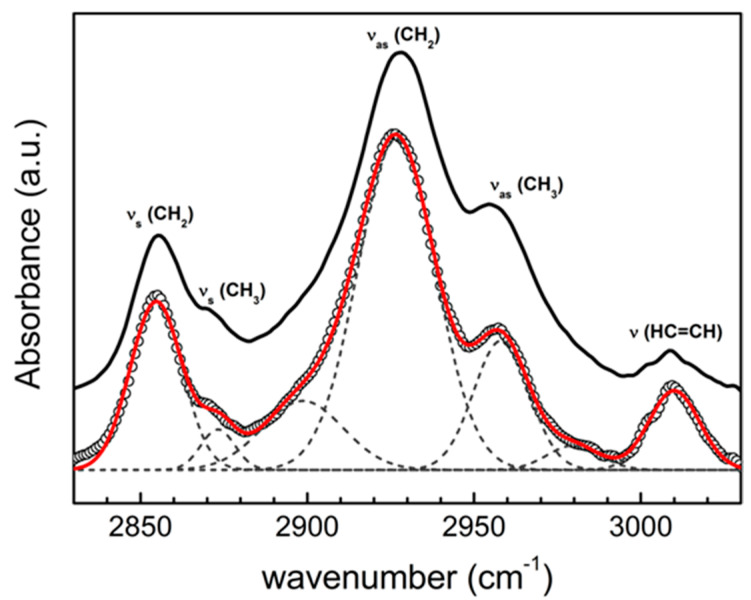
ATR-IR spectrum of large unilamellar vesicles (LUVs) dispersed in PBS buffer (empty points), deconvolved with model functions (dashed lines) and resultant fit curve (red). Comparison with ATR-IR spectrum of cyt c-LUV system (black curve) is reported for sake of clarity (*ρ* = 0.17).

The CH vibrational spectrum of CL-containing LUVs is reported in Figure 1. The most intense features were fitted with model functions (see Section 4) in order to assign the specific vibrational modes. The two components at 2855 cm^−1^ and at 2873 cm^−1^ are assigned to the ν_s_ (CH_2_) and ν_s_ (CH_3_) symmetric stretching, respectively; the most intense contribution at 2927 cm^−1^ is ascribed to the asymmetric stretching ν_as_ (CH_2_), while the one at 2956 cm^−1^ to the ν_as_ (CH_3_) stretching vibration. The high frequency peak at 3012 cm^−1^ is attributed to the stretching mode of the olefinic HC=CH group. Less intense contributions, observed as sidebands around 2900 and 2980 cm^−1^, are ascribed to mixed stretching modes of the acyl chains.

**Figure 2:**
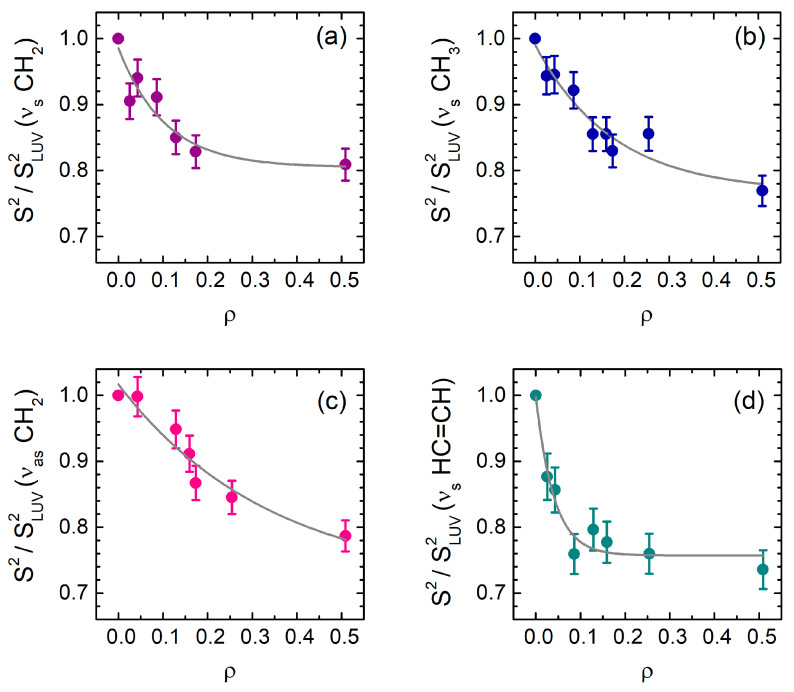
*S*^2^ values for the principal spectral bands: (**a**) ν_s_ (CH_2_) at 2855 cm^−1^, (**b**) ν_s_ (CH_3_) at 2873 cm^−1^, (**c**) ν_as_ (CH_2_) at 2927 cm^−1^, and (**d**) ν (CH=CH) at 3012 cm^−1^ peaks, as a function of *ρ*, normalized to the value obtained from the pure LUV spectrum, with exponential fit of the data.

We first focused on the spectral weights (*S*^2^, see Materials and Methods) of the CH stretching vibrations, as they provide information on the lipid membrane changes and on the affinity of the protein-membrane binding. Normalized *S*^2^ values of the ν_s_ (CH_2_), ν_s_ (CH_3_), and ν_as_ (CH_2_) modes are reported in Figure 2a–c respectively; the *S*^2^ of the olefinic mode at 3012 cm^−1^ is reported in Figure 2d. In all cases, a decrease of the intensity by increasing *ρ* is observed, with a total relative reduction that varies from ≈−20% to ≈−30%. However, the steepness of each curve is different, and the asymptotic limit is indeed reached at distinct *ρ* values, suggesting the occurrence of multiple effects. The intensity of the two CH_2_ modes and of the CH_3_ band is characterized by a multiexponential smooth decrease, while the *S*^2^ values of the olefinic band at 3012 cm^−1^ reach the plateau very quickly, already at *ρ* ≤ 0.2, with a single exponential trend (Figure 2d). Previous studies have demonstrated that, upon binding to cyt c, one CL acyl chain can be inserted into a protein hydrophobic cavity [11], thus stabilizing the protein–membrane interaction. The values of protein concentration at which such process has been observed overlap the same range of protein/lipid ratios reported in Figure 2d (i.e., 0 < *ρ* < 0.2), suggesting a possible rationale for the decrease of the *S*^2^ intensity of the CH=CH vibrations relative to the CL tails. 

**Figure 3:**
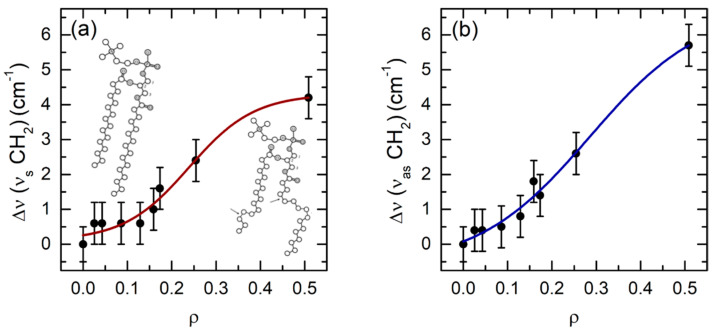
Frequency shift Δν (in cm^−1^) as a function of *ρ*: (**a**) peak at 2855 cm^−1^, ascribed to CH_2_ symmetric stretching mode, with sketches of trans/gauche CL conformers; (**b**) peak at 2927 cm^−1^, assigned to CH_2_ antisymmetric stretching mode. Data were fitted by sigmoidal curves (shown as guides to the eye) and reported on the same scale.

In order to better characterize the effects induced by cyt c binding on the lipid order, changes in the vibrational frequencies have also been taken into account, evaluating the peak frequency from fits to the data and calculating the frequency shift as Δν = ν*^ρ^* − ν*^LUV^*, where the superscript *ρ* refers to a given *ρ* value. Within the experimental error, no shifts were observed for the CH_3_ and the HC=CH bands (data not shown); in contrast, considerable effects were produced on the frequency of both symmetric and asymmetric stretching modes of the CH_2_ groups. These data, reported in Figure 3, are described by sigmoidal trends, with significative changes starting from *ρ* ≥ 0.1. A shift towards higher frequencies of the CH_2_ bands has been generally associated to the loosening of membrane rigidity [22], an occurrence that does not rule out conformational changes of the acyl chains at the molecular level. Indeed, it has also been reported that the hardening of the CH_2_ stretching modes may be related to an increase in the number of gauche conformers: This effect was observed in the case of bilayer hydration, which leads to the activation of torsional motions of acyl chains, typical of the liquid-crystalline phase [23]. Therefore, the trends observed in Figure 3a,b suggest that protein binding produces, at high *ρ* values, a number of disordered structures within the lipid bilayer.

#### 2.1.2. Spectra of the LUV Polar Heads

**Figure 4:**
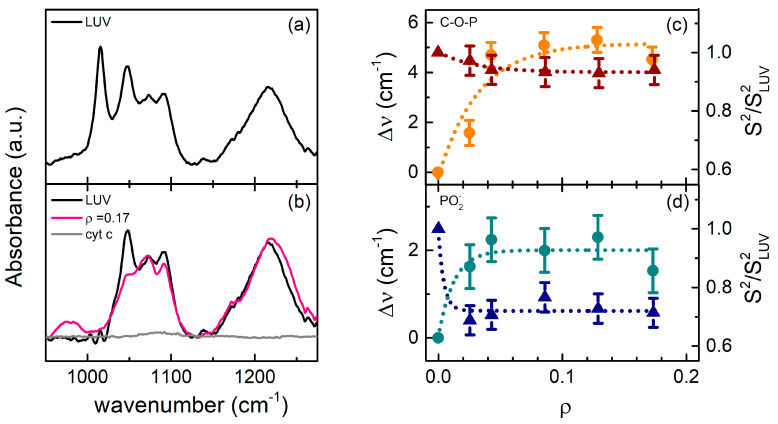
(**a**) ATR-IR spectrum of freshly prepared LUV solution; (**b**) ATR-IR spectra of LUV before (black) and after (pink) cyt c interaction, compared to the spectrum of the pure cyt c (gray). Spectra were normalized to unit area; (**c**) C-O-P Δν frequency shift (orange circles) and *S*^2^ values (dark red triangles) normalized to those of LUV solutions as a function of *ρ*; (**d**) ${\mathrm{PO}}_{2}^{-}$ Δν frequency shift (cyan circles) and normalized *S*^2^ values (blue triangles) of ${\mathrm{PO}}_{2}^{-}$ groups. In (**c**,**d**) panels, data were fitted with exponential curves, reported as dotted guides to eyes.

At variance with the signal of the acyl chain constituents (CH_2_, CH_3_, and CH=CH), the ATR-IR bands of the CL phosphate groups are particularly sensitive to changes occurring at the membrane surface. ATR-IR spectra in the region of the polar heads are reported in Figure 4a,b. First of all, an intense contribution centered at 1015 cm^−1^ was observed in selected native LUV solutions (Figure 4a). It is worth mentioning that a similar spectral feature was observed around 1027 cm^−1^ in dipalmitoyl-phosphatidyl-choline (DPPC) and assigned to N-C-C modes of the polar head [24]: its presence has been considered a signature of the lipid molecule integrity. A straightforward scaling of the peak frequency, considering the substitution of N atom with one glycerol oxygen, suggests the same origin for the 1015 cm^−1^ band detected in CL, also in agreement with previous experimental results [25]. In this context, the 1015 cm^−1^ absorption can be assumed as a spectroscopic marker of the healthiness of the CL molecules within LUVs.

Two other relevant features are observed in the spectrum of native LUV solution (Figure 4a): a manifold of peaks between 950–1100 cm^−1^ and a large band centered at 1225 cm^−1^. The latter is assigned to the asymmetric stretching of the O-P-O^-^ group, ν_as_ (${\mathrm{PO}}_{2}^{-}$), while the manifold at the lowest frequencies comprises both the symmetric O-P-O^-^ stretching, ν_s_ (${\mathrm{PO}}_{2}^{-}$), as well as the stretching modes of phosphate ester (C-O-P) groups [25,26,27]. Band assignment, based on well establish infrared data, ascribes the 1050 and 1070 cm^−1^ absorptions to the phosphate ester vibrational modes, and that at 1090 cm^−1^ to the ν_s_ (${\mathrm{PO}}_{2}^{-}$). Moreover, sidebands are observed around 970 cm^−1^, associated to ν (${\mathrm{PO}}_{3}^{-}$) modes, and at 1175 cm^−1^, ascribed to the CO-O-C carboxyl modes. Spectra collected at increasing *ρ* values show frequency shifts and change in intensity of the ν_s_ (${\mathrm{PO}}_{2}^{-}$) and ν (C-O-P) bands, as reported in Figure 4c,d. It is well known that the frequencies of the P=O modes strongly depend on the CL hydrated state, since hydrogen bonds with solvent shift these absorptions to lower frequencies [26,28]. Recently, it has been demonstrated by an electrochemistry approach that the adsorption of cyt c can be contrasted, inducing a structured solvation shell at a membrane surface [29]. Therefore, the observed small hardening of the phosphate group frequencies reported in Figure 4c,d endorses the hypothesis of a substitution of tightly hydrogen bonded water molecules with softer cyt-CL interactions, most likely of hydrophobic character. 

As regards the absorption intensities, data in Figure 4c,d show a decrease in both C-O-P and ${\mathrm{PO}}_{2}^{-}$ modes, particularly relevant (≈−30%) in the latter ones. Such an intense drop can be induced by a major constraint of these groups in protein binding, resulting in phosphate conformers with low oscillator strength.

### 2.2. Fluorescence Spectroscopy

As suggested by the above reported ATR-IR measurements, only minor changes occur in the membrane structure when the number of protein molecules interacting with the lipid double layer is very small (for instance *ρ* ≤ 0.04). In order to get complementary information on the early events characterizing cyt c association to the lipid vesicles, we used Fluorescence Resonance Energy Transfer (FRET), a particularly suitable technique to monitor interactions between macromolecules. Indeed, different kinds of FRET-based experiments have been successfully carried out in the past to investigate the cyt c binding to synthetic vesicles. In one type of measurement (type 1) [8,9,10], the number of cyt c molecules has been kept constant varying the lipid concentration; in a second kind of experiments (type 2), the binding has been instead monitored increasing the number of protein molecules, at constant vesicle concentration [7,11,17,30]. 

**Figure 5:**
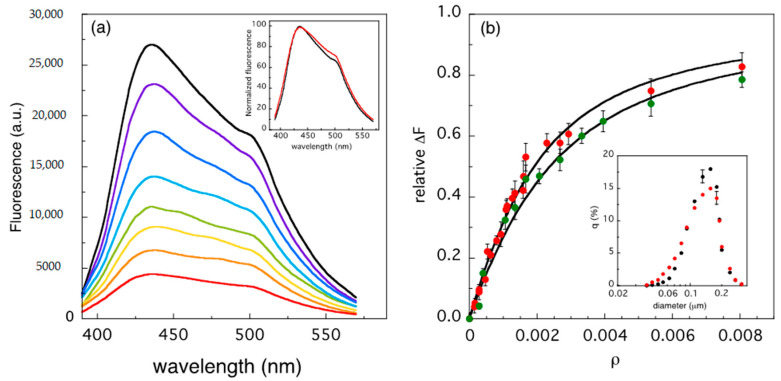
(**a**) Laurdan fluorescence as a function of *ρ* (black line: *ρ* = 0; red line: *ρ* = 0.008); in the inset, the initial (black) and final (red) normalized spectra are reported; (**b**) normalized total fluorescence change of wild type-cyt c (red) and K72N-mutate cyt c (green) as a function of *ρ*. Solid lines correspond to the best fits (described in Section 4), which yield the dissociation constant values K_d WT_ = (0.18 ± 0.03)·10^−6^ M and K_d K72N_ = (0.24 ± 0.03)·10^−6^ M, and the number of lipids interacting with each protein molecule N_L WT_ = (21 ± 7) and N_L K72N_ = (24 ± 8), respectively. In the inset, the size distribution of the LUVs obtained by light scattering is reported, in the absence (black points) and in the presence (red points) of wt-cyt c. A representative error bar has been included in each dataset.

We carried out this second kind of studies using laurdan molecules in the lipid bilayer as donors and the protein heme groups as acceptors. The dependence of the laurdan spectrum on the protein concentration is reported in Figure 5a, as a function of *ρ* values. The relative fluorescence change (ΔF/F_Max_) obtained from these spectra (see Materials and Methods) is reported in Figure 5b. The data show a high affinity of cyt c for the LUVs, in agreement with what already observed in other binding experiments carried out with 100% CL-containing vesicles [10,31]. The comparison between the normalized spectra at *ρ* = 0 and *ρ* = 0.008 reveals the occurrence of a small red-shift in the presence of cyt c (inset of Figure 5a). The shape of the laurdan spectrum is known to reflect lipid order in synthetic vesicles [32] and packing in natural membranes [33]. Such features can be conveniently described by the so-called general polarization (GP), parametrized by an index in the interval -1 and +1: lowest negative values correspond to liquid-crystalline states, while the highest are typical of gel phase [32]. The GP values calculated for the spectra at *ρ* = 0 and *ρ* ≈ 0.008 are 0.19 and 0.16, respectively, diagnostic of a marginally ordered bilayer, which undergoes to limited, but yet appreciable, effects upon cyt c binding. 

In this regard, we also controlled the integrity of the LUVs at different *ρ* values, in a parallel set of Dynamic Light Scattering (DLS) experiments. The inset of Figure 5b shows that the size distributions of the LUVs, in absence and in presence of cyt c, are superimposable, thus ruling out possible protein-induced damages to the membrane. One strong experimental evidence in favor of the so-called lipid anchorage binding model is the perturbation produced in the cyt c Circular Dichroism (CD) spectrum by the penetration of one phospholipid acyl chain in a protein pocket localized in proximity of the heme group [11]. This mechanism of association involves one lysine residues of cyt c, namely LYS72, as demonstrated using a single point mutated protein, K72N, lacking this amino acid [8]. Indeed, at variance with the wild type (wt)-protein, the CD spectrum of K72N is not affected by the presence of liposomes, thus indicating the failure of CL recognition from the cyt c variant [8]. We took advantage of these findings, repeating the fluorescence measurements using the K72N mutant-protein. FRET data and fit, reported in Figure 5b, demonstrate that at such low *ρ* values no differences between the mutant and the wt-protein is observed. This result suggests that the binding process at low cyt c/lipid ratio must be ascribed to a mechanism that, at this stage (*ρ* ≤ 0.02), does not involve a lipid anchorage mechanism.

## 3. Discussion

Since the early systematic studies on the interaction between cyt c and model membranes, it has been clear that the process is quite complex, depending on several parameters such as pH, ionic strength, and membrane composition [7,31]. The presence of CL within the double layer plays a major role in modulating the association of cyt c to synthetic vesicles, reaching a maximum effect in 100% CL-containing liposomes [31]. Despite the process is rather fast (few minutes) [19], the mechanism of binding is not trivial at all, because multiple microscopic events occur at the membrane surface, strictly depending on the number of involved protein molecules [14,30]. In the last decades, the binding measurements, carried out with several methodologies, yielded two sets of quite different values for the dissociation/association binding constant [34], ranging from K_d_ ≤ 0.4·10^−6^ M [17,30,35] to K_d_ ≈ 5–40·10^−6^ M [9,30,36]. The respective binding curves from which such parameters have been extrapolated approach their asymptotic values at very different *ρ* values. Such a widespread range in part depends on the way the binding experiments are performed, i.e., increasing the vesicle concentration to a solution containing cyt c, or progressively adding cyt c to a fixed number of liposomes. Especially in this second kind of measurements (type 2), when 100% CL-containing liposomes are used, the binding process seems to be completed at very low protein concentration, namely at *ρ* ≈ 0.015–0.020 [9]. Our results yield quite a similar value: according to the parameters obtained by the non-linear fit, the 95% saturation of the curves reported in Figure 5 is reached at *ρ* ≈ 0.023. Conversely, in CD measurements (typically belonging to type 1 experiments), the effects on the protein tertiary structure are detected at *ρ* ≈ 0.2, with vesicle surface strongly crowded by cyt c molecules [11,36,37]. Our ATR-IR measurements reflect such complexity and state for a complementary approach, which accounts for the structural and conformational changes of the lipid network upon cyt c binding. In particular, the results reported in Figure 2, Figure 3 and Figure 4 provide evidence that protein crowding produces different kind of perturbations on the double layer structure, in three specific protein concentration ranges. 

The first outcome occurs at *ρ* ≤ 0.02, with a small number of cyt c molecules interacting with the membrane. As shown in Figure 4d, perturbations on the ${\mathrm{PO}}_{2}^{-}$ vibrational frequencies indicate that the earliest binding process mainly involves the polar heads of CL molecules. This finding agrees with the results obtained by Muenzner and co-workers [38], who observed a peripheral binding of cyt c to 50% CL-containing liposomes, at very low protein concentrations (*ρ* < 0.001). In that study, no evidence of protein-membrane association occurring through the insertion of a lipid acyl chain in the cyt c hydrophobic core was noticed. This insertion has been indeed observed at much higher *ρ* values, typically at *ρ* ≥ 0.15 [11,12,36]. The higher propensity of CL for the inner shell of mixed phospholipid bilayers [39], as well as the entropic cost of chain reversal (from the outer membrane leaflet), suggests that lipid anchorage is a rare occurrence at low protein concentration. Furthermore, the almost identical FRET data of wt- and K72N mutant-cyt c, shown in Figure 5b, confirm this picture, and indicate that the binding occurs with a high affinity (low dissociation constant), as reported by previous works [17,30]. 

The cationic nature of cyt c and the strong negative charge of CL-containing vesicles suggest an early interaction of the protein with the double layer of electrostatic nature, which has been found to be strictly dependent on the CL content, being particularly evident in the case of 100% CL-containing liposomes [10]. Further, the initial electrostatic interaction has been addressed to a lysine-rich cyt c domain (the so-called A site) [14,30,31]. On the other hand, a second mechanism of association has also been proposed [31], mainly involving hydrogen bonds and hydrophobic interactions, in a different cyt c domain (site C). The changes observed in intensity and frequency of CL phosphate modes (Figure 4c,d) attest that substitution of solvent molecules with the protein peripheral side chains occurs at the membrane surface and suggest that important hydrophobic components stabilize the interaction, even at the early stage of the binding process. Such environmental transformation at the membrane surface can be responsible for the conformational change of the protein (from a compact to an extended structure) since its first contact with the double layer [9]. This effect, already observed by single-molecule fluorescence correlation spectroscopy [38], has been tentatively proposed to characterize the binding kinetic of cyt c [10,38]. According to this model, the protein-membrane association rapidly switches from an initial electrostatic-driven process to a hydrophobic-guided interaction [38]. Gorbenko and co-workers estimated that, upon binding, 10–15 lipid molecules are present in the cross-sectional area of cyt c in the membrane plane, using a 10–40% CL content [30]. The fluorescence binding curves reported in Figure 5b reveal that a larger number (≈20) of lipid molecules are engaged in the contact with cyt c, suggesting that a tight association takes place using 100% CL-containing vesicles. A similar result (≈25 lipids per cyt c) was obtained by Rajagopal and co-workers [40]. In their study, they demonstrated that such cyt c/CL stoichiometry is strictly dependent on the electrostatic repulsion among the protein molecules, as a variant lacking a positive charge at the protein surface (namely R91A) contacts a smaller number of lipids (≈15). These findings are quite interesting because an extensive coverage of the membrane increases lateral pressure [14], causing cyt c penetration into the double layer. The electrostatic dependence of the cyt c distribution on the LUV surface requires some consideration on the size and shape of the surfaces involved in the binding process. Assuming 20 lipids per cyt c, a diameter of about 34 Å for a folded protein molecule, and an average CL polar head area of about ≈120 Å^2^, the percentage of cyt c surface involved in the contact with the membrane results to be ≈65%, i.e., more than one half of the whole area of a spherically shaped molecule. A possible explanation could reside in the above mentioned cyt c conformational changes, since a partial loosening of the tertiary structure leads to the exposure of a larger portion of the external surface with respect to a compact and spherical protein molecule and to the re-arrangement of the charged amino acid at the surface. Alternatively, the penetration of cyt c into the lipid double layer can be considered. The data reported in Figure 2 and Figure 5 suggest that, for *ρ* < 0.02, the membrane undergoes minor structural changes, consisting in a small reduction of the CH stretching intensity (Δ*S*^2^ ≈ −4%) and a larger decrease of the laurdan generalized polarization (ΔGP ≈ -15%). The modest size of these effects seems to exclude a deep insertion of cyt c into the membrane; however, both results point in the same direction of a progressive lipid disordering in the presence of cyt c. The possibility that even at very low concentration the protein binding can affect the LUV structure has been recently proposed by Schweitzer-Stenner [34], in order to explain the discrepancy observed in the dissociation constant values measured with different experimental approaches. In such a frame, quite a complex mechanism seems to emerge: on one side, the proximity to CL polar heads induces partial unfolding of cyt c tertiary structure; on the other, the membrane order is slightly compromised, probably facilitating a more incisive protein-membrane interaction. 

Upon increasing the number of protein molecules at the vesicle surface, in the 0.02 ≤ *ρ* ≤ 0.2 range, the *S*^2^ strength of the olefinic group undergoes a sharp transition (Figure 2d), diagnostic of relevant structural changes in the vesicle hydrophobic moiety. As already mentioned, at these protein concentrations, modifications in the heme absorption bands have been observed in the presence of both free oleic acid and phospholipid vesicles, suggesting the occurrence of a lipid-anchorage [11]. Protein crowding at the membrane surface increases the probability of cyt c penetration into the hydrophobic site of the bilayer, enhancing lipid disordering [14,30]. Indeed, a major decrease in the *S*^2^ values of the CH groups occurs for 0.02 ≤ *ρ* ≤ 0.2. (Figure 2a–c), in the same range of the olefinic transition (Figure 2d), attesting the concomitant loosening of the membrane structure. A possible explanation for such behavior can reside in the capacity of cyt c to cluster the CL molecules, thus inducing a negative curvature at the double layer surface, a mechanism already proposed for pore formation, at high protein concentration [20]. 

The existence of a two-step binding mechanism, as predicted in previous works [10,41] and suggested by our FRET and ATR-IR data, has been recently confirmed by Elmer-Dixon and co-workers [42]. In particular, they found that, for *ρ* ≈ 0.04, a partial loosening of the cyt c tertiary structure occurs (as revealed by an increase in tryptophan fluorescence), while at higher protein/lipid ratio (*ρ* ≥ 0.1) the heme environment undergoes important conformational changes (detectable by CD in the Soret spectral region). Interestingly, they stated that such behavior is a peculiarity of the convex outer surface of the liposomes generally used in in vitro binding measurements. The protein association to concave lipid surfaces indeed occurs in a single step process, thus demonstrating that the complex behavior of cyt c depends not only on the protein crowding at the membrane surface, but it is also dictated by the bilayer shape. 

A final set of phenomena occurs at very high protein concentration (*ρ* > 0.2). In this condition, changes in the intensity (Figure 2a–c) and in the frequency of CH_2_ and CH_3_ modes are only detected (Figure 3), diagnostic of major processes leading to loss of the CL acyl chain structure, such as the occurrence of trans-to-gauche transition. Despite the fact that this phenomenon has been extensively studied in lipids as a function of environmental condition [21,43,44,45], only in a few cases it has been used to exploit the protein-lipid interaction [46,47,48]. On the other hand, at such high cyt c concentration, the protein coverage of the vesicles is expected to exert a lateral pressure on the membrane [14], which is known to increase its permeability [49], causing major damages such as pore formation [20] and membrane fusion [35].

**Figure 6:**
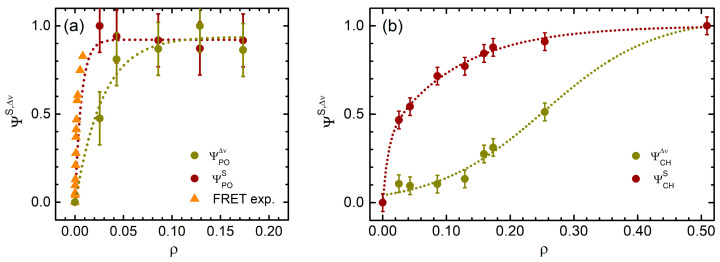
(**a**) Order parameter obtained from the frequency shift Δν (dark yellow) and from the intensities *S*^2^ (dark red) for the phosphate modes, compared to the normalized FRET data, as a function of *ρ*; (**b**) order parameter obtained from the frequency shift Δν (dark yellow) and from the intensities *S*^2^ (dark red) for the hydrocarbon modes as a function of *ρ*. Fits to data are reported as dotted lines as guides to eyes.

All these transformations of the membrane structure can be viewed as a kind of order-to-disorder transition, a perspective that allows a more quantitative analysis, grouping all the results obtained in different classes, concerning the main CH and phosphate spectral properties, respectively. In particular, we introduced two order parameters, defined as the average change in the vibrational frequency, $\Psi^{\Delta \nu }$, and the average decrease in the oscillation strength, $\Psi^{S}$, both normalized between 0 and 1 (Figure 6a,b). In this framework, the fluorescence relative change reported in Figure 5b can also be viewed as an additional, independent order parameter and its values were directly integrated in Figure 6a, as they perfectly overlap the transition observed for $\;\Psi_{PO}^{S}$.

The different behaviors of $\Psi^{\Delta \nu }$ and $\Psi^{S}$ summarize the multiple phases of cyt c binding to the LUVs. In particular, the sudden change of $\Psi_{PO}^{S}$ and in the FRET data (Figure 6a) reflects the initial contact between the protein and the membrane, which is accompanied by the mutual structural changes observed for *ρ* < 0.02. Then, the order parameter associated to the phosphate mode frequencies, $\Psi_{PO}^{\Delta \nu }$, can describe the lipid-driven anchorage of cyt c (Figure 6a), which has been proposed as the main stabilizing factor of the mutual interaction [11,12,36]. Finally, the $\Psi_{CH}^{\Delta \nu }$ and $\Psi_{CH}^{S}$ of the acylic chains moiety account for the major structural changes of the LUVs, characterized by long-range order-disorder transition. It is noteworthy to observe that the two parameters reach the asymptotic value with sigmoidal ($\Psi_{CH}^{\Delta \nu }$) and hyperbolic ($\Psi_{CH}^{S}$) trends, respectively, further highlighting the occurrence of multiple, parallel phenomena.

## 4. Materials and Methods

### 4.1. Materials

Horse heart cyt c (type VI, oxidized form), bovine heart cardiolipin (>80% polyunsatured fatty acid content, primary linoleic acid; 98% purity), and laurdan were provided from Sigma Aldrich- Merck KGaA (Darmstadt, Germany) and used without further purification. All reagents were analytical grade. 

For mutant cyt c the expression plasmids of horse cyt c (pHCyc) [50] was subjected to one round of mutagenesis with QuikChange Site-Directed Mutagenesis Kit (Agilent Technologies, Santa Clara United States), which introduced Lys72Asn substitution into the horse cyt c gene. Mutant pHCyc plasmids was introduced into *E. coli* JM 109. Protein expression and purification of the recombinant protein were then conducted as previously described [51].

### 4.2. Sample Preparation

Aqueous dispersions of CL liposomes were prepared as follows: a film of lipid was prepared on the inside wall of a round bottom flask, by evaporation of a chloroform solution containing the proper amounts of lipid (3 mg). The films obtained were stored in a desiccator overnight under reduced pressure, then 1 mL of PBS buffer solution (pH 7.4) was added to obtain a 2.0 mM lipid dispersion. Solutions were vortex-mixed and then freeze-thawed six times from liquid nitrogen to 30 °C. Dispersions were then extruded (10 times) through a 100 nm polycarbonate membrane. Extrusions were carried out at 30 °C. The vesicle size was determined by light scattering measurements using a Horiba LB550 (Kyoto, Japan) nanoparticle size analyzer. In the buffer, the vesicle diameter was approximately 130 nm. For FRET experiments, liposomes were prepared adding at the initial stage laurdan (17 mM) to the flask with cardiolipin.

In ATR-IR and FRET experiments, lipids concentration was kept constant (equal to 1.2 mM and 0.6 mM, respectively) and measurements were performed adding small amounts of concentrated cyt c (wild type or mutant) in order to obtain different samples with the desired *ρ*.

### 4.3. ATR-IR Experiments and Data Analysis

ATR-IR measurements were performed at the DAΦNE-Light INFN-LNF facility, by using a Vertex 70 V spectrometer (Bruker, Billerica, MA, USA) equipped with liquid nitrogen cooled HgCdTe detector and with ATR device working in vacuum. Measurements were collected in the range 650–4000 cm^−1^ with 2 cm^−1^ resolution, coadding 128 interferograms. Correction of the spectra for the refractive index of the ATR crystal was performed by software. Experiments were performed at least threefold, to confirm and optimize the results. In each experiment, the spectrum of cyt c solution 1.2 mM was previously collected and appropriately scaled and subtracted to those of the cyt c-LUVs solutions once the protein concentration differences were accounted by a fit in the Amide I-II region (1550–1700 cm^−1^). Spectra were baselined with a polynomial curve and then normalized to unit area, to ascribe the absorption features to the single CL molecule. In the phosphate spectral region, selected spectra showed the band centered at 1015 cm^−1^, which was fitted with a Lorentzian curve (see Figure 4b) and subtracted before the evaluation of the spectral contributions. 

Spectral contributions were then fitted with Lorentzian functions:

(1) A(ν)=S2π Γ2(ν−ν0)2+(Γ2)2

which returned the strength of the vibrational modes *S*^2^, the linewidths Γ, and the peak frequencies ν_0_. The amplitude of the Lorentzian peak is related to the number of oscillators *n*, the effective dipole charge *q_eff_*, and mass *m_eff_* through:

(2) $$S^{2}\propto \frac{nq_{eff}^{2}}{\varepsilon \;m_{eff}}$$

being *ε* the dielectric function of the medium [52]. *S*^2^ and Δν values reported in the Results were obtained as average values of *N* repeated measurements; their errors were estimated as the standard deviation divided by $\sqrt{N}.$

### 4.4. Fluorescence Measurements and Data Analysis

The binding of cyt c to LUVs was monitored through FRET measurements between laurdan molecules incorporated in the liposomes and the cyt c heme group. The laurdan spectra (λ_ex_ = 380 nm, λ_em_ = 390–560 nm) were collected on a K2-ISS (ISS, Inc., Champaign, IL, USA) as a function of *ρ*. A correction for inner filter effect has been taken into account [17], introducing (at each protein concentration) the multiplicative correction factor:

(3) $$K=10^{\frac{A\left(em\right)+A\left(ex\right)}{2}}$$

where *A*(*em*) and *A*(*ex*) are the absorption at the emission and excitation wavelengths, respectively. A further experimental control consisting in a standard procedure (used for instance in fluorescence quenching measurements [53]) yielded a similar correction curve. Briefly, the fluorescence of laurdan was observed placing two further empty cuvettes in the excitation and emission pathways of the fluorometer, i.e., before and after the sample holder. Then, the two cuvettes were filled with buffered solution containing increasing cyt c concentrations and the fluorescence of laurdan measured again, obtaining the correction required. The Δ*F* = *F*_0_ − *F_ρ_* difference between the area at *ρ* = 0, *F*_0_, and those collected at increasing *ρ* values, *F_ρ_*, was plotted and interpolated with a hyperbolic function, obtaining the asymptotic value, Δ*F_max_*. The relative fluorescence change Δ*F_re_*_l_ = Δ*F*/Δ*F*_max_, reported in Figure 5b, was fitted using a variant of the theoretical approach developed by Hille and co-workers [54] and used in the past for other membrane-binding proteins [55,56,57]. In particular, the equilibrium between the free protein *P_F_* and the number *δ* [L_F_] of possible interacting lipids was considered:

(4) $$P_{F}+\;\delta \;\left[L_{F}\right]↔\left[L_{B}\right]\;\mathrm{with}\;K_{D}=\frac{\left[P_{F}\right]\;\delta \;\left[L_{F}\right]}{\left[L_{B}\right]}$$

where K_D_ is the dissociation constant, [L_F_] and [L_B_] are the concentrations of free and bound lipids, and δ (≪0.5) is the percentage of those observable upon binding, i.e., those of the external leaflet of the LUVs and, among them, the fraction within a distance of about 20 Å far apart from the laurdan molecule (i.e., the minimum Forster energy transfer radius [58]). 

Considering that $\delta \;\left[L_{F}\right]=\delta \;\left[L_{tot}\right]-\left[L_{B}\right]$, and assuming that the relative fluorescence change of laurdan is proportional to the concentration of bound lipids (Δ*F_rel_* ≈ [L_B_]), the expression of the fitting function results to be:

(5) $$\Delta F_{rel}=\frac{A}{2\;\delta^{2}\;\left[L_{tot}\right]}-\sqrt{\frac{A^{2}-4\;N\;\delta^{3}\rho \;{\left[L_{tot}\right]}^{2}}{4\;\delta^{4}\;{\left[L_{tot}\right]}^{2}}}$$

(6) $$\mathrm{with}\;A=N\;\delta \;K_{D}+N\;\delta \;\left[L_{tot}\right]\;\rho +\;\delta^{2}\;\left[L_{tot}\right]$$

where N is the average number of lipid “hidden” by cyt c association with the bilayer. 

## 5. Conclusions and Future Perspectives

The ATR-IR and FRET datasets report on a series of very heterogeneous events driven by cyt c interaction with membranes. Such a complex behavior arises from the mutual structural transformations occurring at the liposome external surface when the protein contacts and hides the CL polar heads from the solvent molecules. The progressive loosening of the lipid structure can be viewed in terms of order-to-disorder transition, with parameters that can quantitatively describe the different steps of the process (Figure 6). 

Despite the advances in this field using model vesicles, an important biological feature must be taken into account in future studies, namely the peculiar shape of the mitochondria inner membrane. As recently found, the bilayer curvature plays a fundamental structural role in CL-enriched vesicles, and dramatically affect the cyt c binding process [42], thus suggesting that not only the composition but also the geometry and size of the bilayer are fundamental to understand the puzzling in vivo behavior of cyt c.

## Data Availability

Not applicable.

## Abbreviations

CL	cardiolipin
cyt c	cytochrome-c
LUV	Large Unilamellar Vesicle
PBS	Phosphate Buffered Saline
ATR	Attenuated Total Reflectance
IR	Infrared
FRET	Fluorescence Resonance Energy Transfer
GP	general polarization
